# Wastewater-Based Surveillance of SARS-CoV-2 for Early Warning of COVID-19 Infection Dynamics

**DOI:** 10.3390/v18050569

**Published:** 2026-05-18

**Authors:** Qiuyan Zhao, Xinye Zhang, Jing Peng, Xiaoyan Ma, Yongxing Wang, Jun Luo, Xiaohan Su, Siyu Yang, Xiaona Yan, Yuan Wei, Jie Zhang

**Affiliations:** 1 Department of Environmental Sanitation, Henan Provincial Center for Disease Control and Prevention, Zhengzhou 450016, China; hncdczhaoqiuyan@163.com (Q.Z.); blanche2019@foxmail.com (X.Z.); henancdc_pj@163.com (J.P.);; 2 Office of Preventive Medicine Association, Henan Provincial Center for Disease Control and Prevention, Zhengzhou 450016, China; 3 Information Center, Henan Provincial Center for Disease Control and Prevention, Zhengzhou 450016, China

**Keywords:** wastewater surveillance, SARS-CoV-2, COVID-19, generalized additive model, early warning

## Abstract

Wastewater-based epidemiology has emerged as a valuable complementary tool for population-level monitoring. This study evaluated the early warning value of wastewater surveillance for monitoring SARS-CoV-2 and its correlation with COVID-19 infection trends. From May 2024 to December 2025, 526 wastewater samples were collected from five treatment plants. Spearman correlation and a quasi-Poisson generalized additive model (adjusting for wastewater temperature) were used to assess relationships between SARS-CoV-2 RNA concentration, the number of reported cases, and lag associations. Wastewater viral loads (copies/mL) significantly correlated with reported cases. Wastewater temperature was positively correlated with both viral concentrations and case numbers. A significant lagged association was observed for the N gene, with relative risk peaking at a 10-day lag. Although the ORF1ab gene was not significant for most lag periods, its temporal trend was consistent with that of the N gene. Wastewater surveillance of SARS-CoV-2, particularly targeting the N gene, can effectively predict COVID-19 infection dynamics with a 10-day lead time, thereby supporting wastewater surveillance as an early warning tool for public health monitoring.

## 1. Introduction

The COVID-19 pandemic, a global public health emergency triggered by severe acute respiratory syndrome coronavirus 2 (SARS-CoV-2), has now transitioned to a routine surveillance phase [1,2,3]. With the discontinuation of widespread mass nucleic acid testing, traditional surveillance capabilities have diminished [4]. In this context, accumulating evidence underscores wastewater-based epidemiology (WBE) as an indispensable complementary tool for sustaining effective pandemic surveillance [5,6,7,8].

In contrast to conventional clinical diagnostics, WBE offers superior cost-efficiency, operational simplicity, and non-intrusiveness, making it ideal for broad-scale deployment [9]. Currently, more than 70 countries and regions have launched national wastewater surveillance programs to help monitor community COVID-19 prevalence [10]. Numerous prior studies have established a significant association between SARS-CoV-2 concentrations in wastewater and reported COVID-19 cases [11,12]. Moreover, WBE signals can precede increases in reported case numbers by several days to weeks, offering substantial early warning potential [13,14,15]. Notably, emerging evidence indicates that wastewater physicochemical parameters such as temperature, pH, and total suspended solids (TSS) significantly modulate viral RNA stability, thereby introducing potential biases that complicate data interpretation [16,17].

To evaluate the utility of urban wastewater surveillance for forecasting COVID-19 prevalence, we selected Zhengzhou City, Henan Province, China, as the study area and developed a quasi-Poisson generalized additive model (GAM) to assess the association between viral RNA load and the daily number of reported COVID-19 cases while controlling for wastewater parameters and other potential factors. The findings are intended to provide scientific support for developing a regional early warning system based on environmental surveillance and for strengthening public health responses to emerging infectious diseases.

## 2. Materials and Methods

### 2.1. Wastewater Sample Collection

A total of 526 wastewater samples were collected from the influents of five Zhengzhou municipal wastewater treatment plants (WWTPs) over the study period. These wastewater samples were 24 h composite samples composed of subsamples collected at 1 h intervals using automated refrigerated samplers (Model WF-2020A, Beijing Wondfo Intelligent Technology, Beijing, China). The instrument is equipped with a built-in refrigeration system, and all subsamples were continuously maintained at 4 °C in the sampler throughout the sampling period. Samples were collected once or twice a week between May 2024 and December 2025.

### 2.2. Wastewater Physicochemical Parameters Analysis

Daily wastewater treatment volume (WTV), wastewater temperature, chemical oxygen demand (CODcr), TSS, pH, and ammonia nitrogen (NH_3_-N) were routinely monitored by WWTPs. All physicochemical parameters were determined according to national standard methods as follows: WTV was measured by an electromagnetic flowmeter (HJ/T 367-2007: Technical requirement for environmental protection product–Electromagnetic pipe flowmeters) [18]; water temperature was measured by thermometer or reversing thermometer method (GB/T 13195-1991: Water quality–Determination of water temperature–Thermometer or reversing thermometer method) [19]; CODcr was determined by the dichromate method (HJ 828-2017: Water quality–Determination of the chemical oxygen demand–Dichromate method) [20]; TSS was analyzed by the gravimetric method (GB/T 11901-1989: Water quality–Determination of suspended substance–Gravimetric method) [21]; pH was measured by the glass electrode method (GB/T 6920-1986: Water quality–Determination of pH value–Glass electrode method) [22]; NH_3_-N was determined using Nessler’s reagent spectrophotometry (HJ 535-2009: Water quality–Determination of ammonia nitrogen–Nessler’s reagent spectrophotometry) [23].

### 2.3. Viral RNA Detection

Following the Technical Specification for Surveillance of SARS-CoV-2 in Wastewater (WS/T 10042-2025), all samples were transferred to the laboratory at 0–4 °C and tested within 24 h after collection [24]. Viral concentration was performed by polyethylene glycol (PEG) precipitation. Briefly, 40 mL of wastewater supernatant was mixed with 4.0 g of PEG 8000 and 0.8 g of NaCl (Sangon Biotech, Shanghai, China). Then, the mixture was centrifuged at 12,000 × *g* for 2 h and concentrated to a final volume of 0.5 mL. Afterwards, 0.2 mL of the concentrate was used for total RNA extraction, yielding a final elution volume of 0.06 mL using a commercial magnetic bead-based RNA extraction kit (Tianlong Technology, Xi’an, China).

SARS-CoV-2 RNA was detected by reverse transcription quantitative PCR (RT-qPCR) targeting the ORF1ab and N genes. A fluorescent PCR kit (Suzhou, China) on a CFX96 real-time PCR system (Bio-Rad, Hercules, CA, USA) was used for the amplification. RT-qPCR was performed in a total volume of 25 µL per well. For each reaction, 20 µL of master mix (containing 7.5 µL nucleic acid amplification buffer, 5.0 µL enzyme mix, 4.0 µL viral reaction mix, and 3.5 µL RNase-free water) was combined with 5 µL of extracted nucleic acid template. The thermocycling schedule used to run the plates was 50 °C for 10 min, 97 °C for 1 min, and 45 cycles that alternated between 97 °C for 5 s and 58 °C for 30 s. Ten-fold serial dilutions of a certified SARS-CoV-2 RNA reference material (GBW(E) 091090) were used in triplicate to create a standard curve for absolute quantification. Only when the amplification efficiency fell between 90% and 110% and the coefficient of determination (*R*^2^) was greater than 0.99 were standard curves acceptable. LOD was determined to be 1 copy/mL by testing pseudovirus dilutions (0–10^3^ copies/reaction) in 20 replicates per level, defined as the minimal concentration yielding ≥95% positivity. A sample was considered positive if the amplification curves exhibited a typical S-shaped pattern, and for each of the target genes (N and ORF1ab), at least two out of three replicates had Ct values below 40; the Ct value was then used for absolute quantification by interpolation from the standard curve. Otherwise, the sample was judged as negative. All samples were analyzed in triplicate. The standard deviations of the triplicate Ct values were all less than 0.3. Viral RNA concentrations were expressed as copies per milliliter (copies/mL) of raw wastewater. Every extraction batch contained blank reagent controls to evaluate potential contamination. In order to verify the absence of inhibitory substances during extraction and amplification, process controls spiked with known pseudovirus concentrations (Ruifengkang Technology, Jinhua, China) were processed alongside test samples. To prevent cross-contamination, nucleic acid preparation and amplification were performed in physically separated work areas.

### 2.4. Data on COVID-19 Cases

Data on COVID-19 cases in Zhengzhou in the same period were obtained from the Chinese Disease Prevention and Control Information System, a national internal disease surveillance platform [25]. These data include details such as gender, date of birth, age, date of onset, current residential address, and case classification. Daily reported COVID-19 cases were aggregated by symptom onset date for comparison with wastewater viral load trends.

### 2.5. Statistical Analysis

The daily normalization of SARS-CoV-2 (which targets the ORF1ab and N genes) in wastewater was calculated as the total of the virus concentration multiplied by the daily WTV at each WWTP on the same day, divided by the sum of the daily WTV across all WWTPs [10,26]. To account for the differences in temporal resolution, the COVID-19 case data were smoothed using 3-day and 7-day rolling averages. The daily weighted average concentration of SARS-CoV-2 data and the daily, 3-day, and 7-day rolling COVID-19 case averages were matched according to the date of wastewater sample collection.

R software (version 4.3.2) was used for all analyses. Spearman correlation was used to evaluate relationships between the concentrations of SARS-CoV-2 RNA in wastewater, the number of COVID-19 cases, and wastewater characteristics. In this work, the relationship between viral concentration and COVID-19 case counts was further quantified using a quasi-Poisson GAM. Wastewater viral load data were log10-transformed to increase comparability and analytical stability. The degrees of freedom (df) for the smoothers were determined using generalized cross-validation as implemented in the R mgcv package. The GAM was specified as follows:

logE(Y*_t_*) = α + *β*X*_t_* + s(*time*, *df* = 7/year) + s(*TempW*, *df* = 5)

where E(Y*_t_*) represents the expected number of reported COVID-19 cases on day *t*; α is the intercept; *β* is the regression coefficient for X*_t_*, denoting the log relative risk (RR) of COVID-19 cases associated with per unit increase in wastewater viral concentration; X*_t_* is the log10-transformed concentration of the SARS-CoV-2 RNA in wastewater on day *t*; s denotes the spline smoothing function; *time* is the date variable with a *df* of 7 per year to adjust for long-term trend and seasonality; and *TempW* represents wastewater temperature with *df* = 5.

## 3. Results

### 3.1. Wastewater Parameter Descriptive Statistics and Correlation Analysis

A total of 526 wastewater samples were collected from five WWTPs, including 266 in 2024 and 260 in 2025. WTV varied across the WWTPs, indicating that normalization of SARS-CoV-2 RNA concentrations in wastewater is necessary to ensure accurate quantification. Wastewater temperatures of all WWTPs ranged from 8.3 °C to 31.2 °C, with an average daily value of 23.8 °C. The daily average values of WTV, TSS, NH_3_-N, pH, and CODcr were 211,000 tons/day, 299.8 mg/L, 34.6 mg/L, 7.3, and 329.8 mg/L, respectively (Table 1).

To explore potential confounding factors affecting SARS-CoV-2 RNA levels and epidemiological dynamics, we further analyzed the correlations among wastewater physicochemical parameters, viral RNA concentrations, and daily, 3-day, and 7-day rolling average COVID-19 cases. The findings demonstrated a positive correlation between wastewater temperature and daily, 3-day, and 7-day rolling COVID-19 case averages as well as SARS-CoV-2 RNA concentrations (target genes ORF1ab and N). Furthermore, only weak negative relationships were seen between the quantities of SARS-CoV-2 RNA and NH3-N, pH, and CODcr.

### 3.2. Correlation Between Reported COVID-19 Cases and Viral RNA Concentration

**Figure 1:**
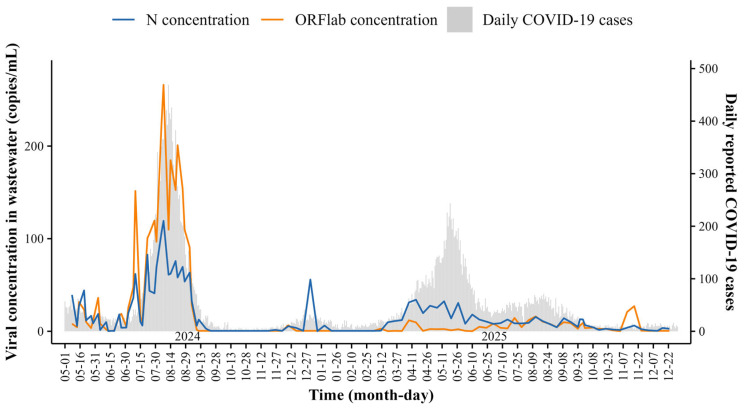
Temporal dynamics of SARS-CoV-2 gene concentrations in wastewater and daily reported COVID-19 cases. The blue line denotes the N gene concentration (copies/mL). The orange line denotes the ORF1ab concentration (copies/mL). The gray bars indicate the daily reported COVID-19 cases.

Based on the data presented in Figure 1, the mean concentration of the ORF1ab gene was 23.31 copies/mL, with values of 44.16 copies/mL in 2024 and 4.37 copies/mL in 2025. The mean concentration of the N gene was 16.66 copies/mL, with values of 23.45 copies/mL in 2024 and 9.23 copies/mL in 2025. During the observation period, 31428 COVID-19 cases—16,003 in 2024 and 15,425 in 2025—were documented. During the study period, peaks in viral RNA concentrations and daily reported COVID-19 cases were observed from July to August 2024 and from April to May 2025, respectively. Outside these periods, both indicators remained at low levels, exhibiting only sporadic, minor fluctuations. The potential of wastewater monitoring as an early warning indication is highlighted by the visual inspection of the time series, which indicates that peaks in wastewater virus concentrations tended to precede or coincide with the peaks in reported cases.

**Figure 2:**
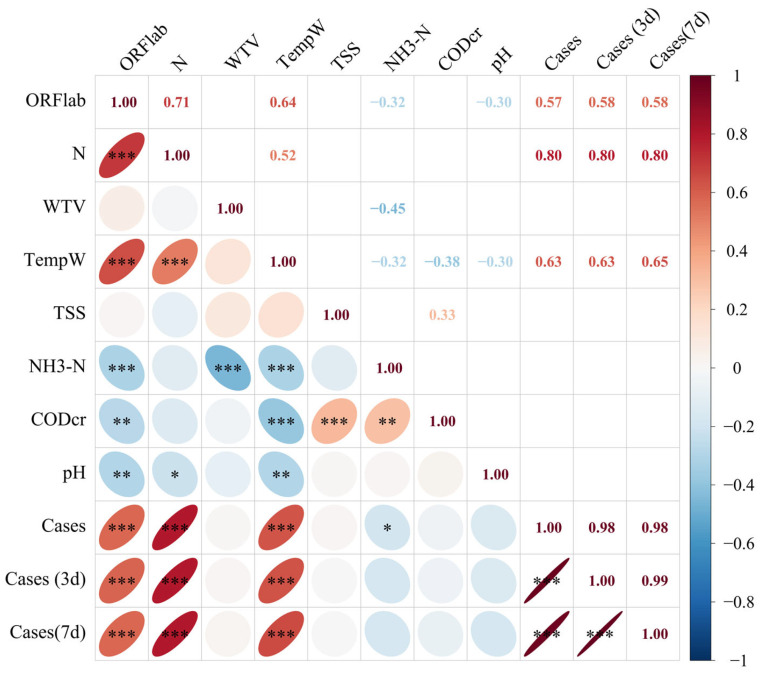
Correlation matrix between SARS-CoV-2 RNA concentrations, wastewater parameters, and reported COVID-19 cases. TempW, wastewater temperature; Cases, daily reported COVID-19 cases; Cases (3 d)/Cases (7 d), 3-day and 7-day rolling case averages. Asterisks denote statistical significance (* *p* < 0.05, ** *p* < 0.01, *** *p* < 0.001).

SARS-CoV-2 RNA concentrations in wastewater and daily reported COVID-19 cases showed a strong positive association, according to Spearman correlation analysis. Both target genes exhibited significant associations with reported COVID-19 cases, though the N gene demonstrated a superior predictive capacity compared to ORF1ab (Figure 2). In particular, the N gene concentration showed a high correlation with the daily cases (*r* = 0.80, *p* < 0.001). Similarly, strong correlations were observed when analyzing 3-day and 7-day rolling case averages (*r* = 0.80 for both, *p* < 0.001). In contrast, while the ORF1ab gene was significantly correlated with daily cases (*r* = 0.57, *p* < 0.001), its association with the 3-day and 7-day rolling averages remained stable (*r* = 0.58 for both, *p* < 0.001). The consistently higher correlation coefficients for the N gene suggest that it is a more stable and reliable indicator for forecasting the number of COVID-19 cases. Additionally, the strong correlations observed between the daily, 3-day, and 7-day rolling case averages indicate that reducing noise by smoothing the data maintains the underlying association, improving the stability of epidemiological models based on wastewater.

### 3.3. GAM-Based Lag Effect Analysis and Case Prediction

**Figure 3:**
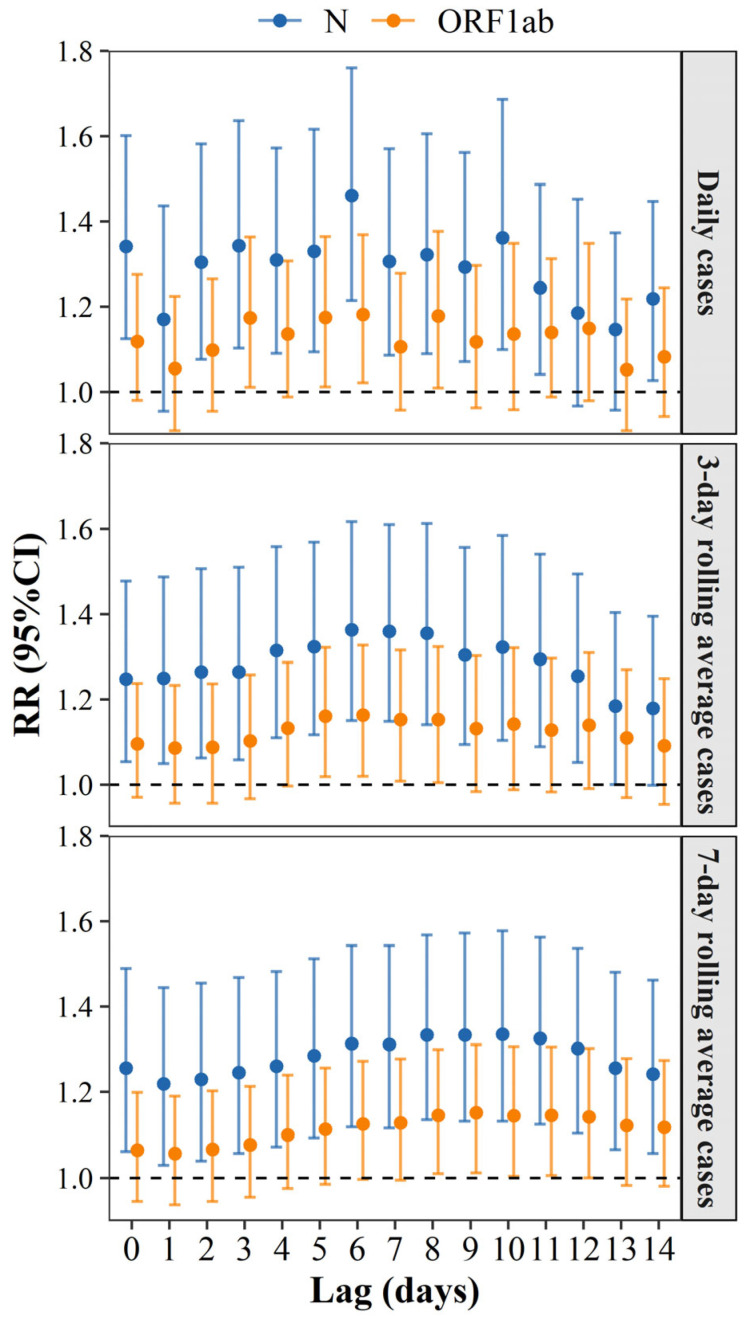
Results from the GAM of SARS-CoV-2 RNA concentration in wastewater against the reported COVID-19 cases. Plotted points represent the RR, and vertical bars indicate the corresponding 95% CIs.

We used GAM to estimate the RR of case increases associated with viral RNA concentrations (log-transformed) at lags ranging from 0 to 14 days to further characterize temporal dynamics and potential leading indicators (Figure 3). Detailed model diagnostic statistics are presented in Figure S1. To evaluate the impact of short-term fluctuations on the stability and interpretability of RR estimates, we analyzed daily case counts alongside 3-day and 7-day rolling averages. In comparison to the daily and 3-day models, the 7-day rolling average model produced the narrowest 95% confidence intervals (CIs).

Furthermore, a distinct difference between the two target genes of SARS-CoV-2 was observed. Across all time scales, the N gene consistently showed greater associations with reported COVID-19 cases than ORF1ab. Within the 7-day rolling average model, elevated viral RNA concentrations during that lag period were consistently associated with increased reported cases. The N gene exhibited statistically significant associations across all lag periods, with a peak association observed at lag day 10. On the other hand, for most lags, the ORF1ab gene did not show any significant relationships. Nevertheless, the overall trends across lag days were consistent between the two genes.

**Figure 4:**
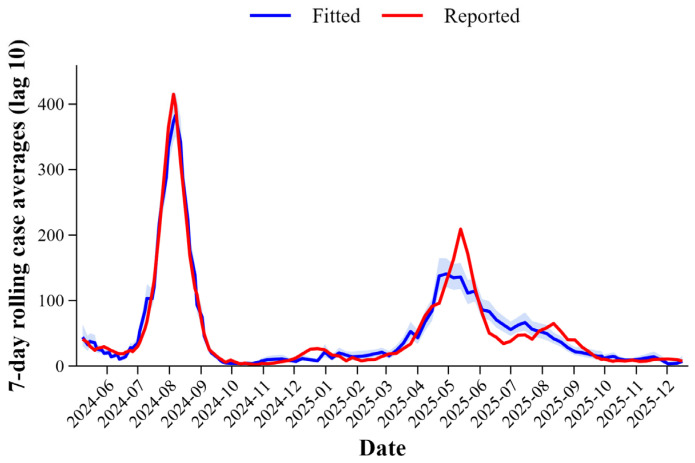
Comparison of reported (red) and model-fitted (blue) 7-day rolling averages of case counts for COVID-19 between May 2024 and December 2025. The fitted values were generated using a GAM with a 10-day lag, where the SARS-CoV-2 N gene concentration measured on day t was used to predict case counts on day t + 10. The shaded region around the blue line represents the 95% CIs for the fitted values.

The ORF1ab gene showed poor predictive usefulness based on the correlation and RR analysis results. As a result, it was left out of later modeling, and only the N gene was kept for further analysis. We used the 7-day rolling average of reported case numbers to reduce temporal noise and improve model stability. Given that the peak RR was identified at a 10-day lag, this interval was included in the final GAM to optimize the early warning lead time. Throughout the study period, fitted case counts from the regression model closely tracked the temporal trends of reported COVID-19 cases, accurately capturing the peaks and troughs of observed incidence data (Figure 4). The plot of fitted against reported case counts indicates that SARS-CoV-2 N gene concentrations serve as an accurate predictor of case data, providing an early warning lead time of 10 days.

## 4. Discussion

WBE represents an evolving frontier in public health surveillance [27]. Serving as a robust early warning system, it offers an unbiased perspective on population health dynamics, effectively complementing traditional clinical data [28]. In this study, we monitored SARS-CoV-2 concentrations in urban wastewater and COVID-19 cases in Zhengzhou during the phase of normalized pandemic prevention and control. Our primary objective was to evaluate the practical utility of wastewater-based surveillance in supporting epidemic prevention and control strategies.

When modeling nonlinear relationships between wastewater viral concentrations, physicochemical parameters, environmental factors, and reported case numbers, GAM is more flexible than conventional linear regression. It also allows for the quantification of lag effects and RR across multiple time windows, thereby better capturing the dynamic and time-lagged characteristics of urban viral transmission [29]. In this study, wastewater temperature was included as a covariate in the GAM. Associations between daily SARS-CoV-2 RNA concentrations and case counts were separately examined using three outcome variables: daily case counts, 3-day rolling averages, and 7-day rolling averages.

Beyond reaffirming the critical utility of WBE for infectious disease surveillance [30], this study elucidates the transmission dynamics of SARS-CoV-2 in Zhengzhou. Consistent with prior research reporting correlation coefficients up to 0.9, we found that temporal fluctuations in wastewater viral RNA concentrations strongly mirrored reported COVID-19 case trends [31,32,33]. Importantly, our analysis revealed differential predictive performance between gene targets: the N gene showed a significantly stronger correlation with case counts (*r* = 0.80, *p* < 0.001) than the ORF1ab gene (*r* = 0.57, *p* < 0.001). This superior performance of the N gene likely stems from its higher genomic copy number per virion and greater stability in wastewater matrices, suggesting it may be a more reliable indicator for monitoring community infection levels in this context [34]. Furthermore, the quantification of the ORF1 gene may be affected by the emergence of novel genetic variants in the viral genome. This divergence could be associated with the reduced amplification efficiency caused by nucleotide sequence alterations in newly circulating SARS-CoV-2 variants, as reported in several previous studies [35,36]. This interpretation is further supported by our monitoring data, which showed that the detection rate of the ORF1ab gene was consistently lower than that of the N gene, reinforcing that the N gene may be a more reliable indicator for monitoring community infection levels in this context. Nevertheless, it is undeniable that, as previous studies have shown, the combination of the N and ORF1ab genes can improve the accuracy and reliability of predictions [26,37].

While prior research has documented correlations between SARS-CoV-2 RNA levels and physicochemical parameters such as COD, total phosphorus, and TSS, our findings present a more nuanced picture [26,38]. Specifically, we observed only weak negative associations with NH_3_-N, pH, and CODcr. Differences from previous findings may be attributable to different wastewater characteristics and the quantity and type of industrial wastewater [39]. Viral behavior in wastewater is influenced by a combination of inactivation, degradation, dispersion, and retardation processes [40]. While water temperature serves as the critical factor influencing viral inactivation, the degradation of viral RNA is mainly attributed to biological processes and chemical reactions [41]. Although the impact of ambient temperature on the COVID-19 transmission rate seems plausible, a definitive conclusion regarding the magnitude of this impact remains unclear [42,43]. Spearman’s correlation analysis indicated a moderately positive correlation between wastewater temperature and both viral RNA concentrations and reported case counts in this study. The observed peaks in COVID-19 cases during August 2024 and May 2025 cannot be attributed solely to ambient temperature fluctuations. Rather, a combination of factors, including the emergence of novel viral variants and potential synergistic interactions with co-circulating respiratory pathogens, likely contributed to these case surges [44]. The elevated viral loads detected in wastewater surveillance during these periods clearly reflected this increased transmission pressure, underscoring the value of WBE as an early indicator of community infection burden. Therefore, to improve the reliability of WBE surveillance, it is imperative to integrate wastewater temperature data into predictive modeling frameworks.

A central finding of this study is the observed lag effect between SARS-CoV-2 RNA concentrations in wastewater and reported case counts. The RR derived from the GAM indicated a statistically significant association between N gene concentrations in wastewater and reported COVID-19 cases. By contrast, the ORF1ab gene did not show a statistically significant association for most lag periods, although its overall temporal trend was consistent with that of the N gene. Furthermore, the use of a 7-day rolling average reduced noise in raw daily case reporting and enhanced model stability, a practice corroborated by earlier research [45,46]. In particular, the RR peaked at a 10-day lag (range 0 to 14 days) according to the study based on the N gene and the 7-day rolling average of reported cases. Multiple studies have reported that SARS-CoV-2 levels in wastewater typically precede surges in COVID-19 cases by approximately 1 to 28 days [47]. For instance, Ansari et al. reported a strong correlation between viral RNA concentrations in wastewater and clinically confirmed cases, with a distinct lead time of up to 14 days [48]. A 2025 study based on wastewater surveillance in Fuzhou further showed that viral loads could predict trends in clinical cases within a 0 to 17-day window, with the strongest correlations identified in certain demographic subgroups [49]. In addition, the large-scale wastewater monitoring program in Hong Kong, using linear regression modeling, indicated that such surveillance could provide a 2- to 4-day lead time for tracking community transmission [50]. This 10-day lead time falls within the range reported by multiple independent studies, further corroborating the predictive value of wastewater surveillance. Mechanistically, viral RNA in wastewater serves as an aggregate signal of catchment-wide shedding, capturing a substantial proportion of undiagnosed and asymptomatic infections. In contrast, clinical case reporting is inherently delayed by the sequential lags spanning from infection to symptom onset, healthcare-seeking behavior, and final diagnostic confirmation [51]. This lead time holds particular importance for routine pandemic prevention and control, offering a critical window for public health responses [48]. Accordingly, this also indirectly suggests that a weekly sampling frequency is feasible for routine surveillance. When an outbreak is detected, the sampling frequency can be increased as needed.

Despite the aforementioned strengths, this study has several limitations. First, we used the weighted average of viral loads from five wastewater treatment plants to represent the overall epidemic situation in Zhengzhou, which constrained the fine spatial resolution of our analysis of COVID-19 transmission patterns. Future studies may attempt to improve the spatial resolution of the model. Second, this study did not incorporate potential confounding variables, including rainfall that may dilute viral concentrations, population mobility, and different viral variants [52]. Third, while a wastewater sampling frequency of one to two times per week effectively captures long-term epidemic trends, it lacks the temporal resolution needed to capture sharp fluctuations and short-term variability in viral concentrations during periods of accelerated transmission. This approach strikes a pragmatic balance between monitoring sensitivity and operational expenses. Consequently, daily sampling is advised during epidemic peaks to more precisely characterize the dynamic evolution of viral loads. Fourth, during the stage of normalized epidemic prevention and control, pandemic fatigue and reduced testing willingness have led to obvious reporting biases, meaning that reported cases represented only a small fraction of the true infections. Such under-ascertainment may cause the model to underestimate the actual transmission potential of the epidemic.

## 5. Conclusions

In summary, this study demonstrates that SARS-CoV-2 wastewater surveillance, specifically targeting the N gene, serves as a reliable early warning tool for COVID-19 in Zhengzhou during the normalization phase. The identified 10-day lead time between wastewater SARS-CoV-2 RNA concentrations and reported cases underscores the value of WBE in bridging the gap between asymptomatic shedding and clinical diagnosis. Additionally, the significant correlation between viral load and wastewater temperature emphasizes the necessity of incorporating physicochemical parameters into prediction models. Our findings strongly support the integration of WBE into routine public health management systems. Future research should optimize monitoring frameworks and integrate comprehensive environmental and demographic covariates to enhance model performance while validating and refining these models across diverse geographic regions and epidemiological contexts.

## Figures and Tables

**Table 1:** Basic Description of Wastewater Parameters. The variability in the measures is expressed as the interquartile range (in brackets).

WWTP Type	n	WTV (10,000 tons/day)	Wastewater Temperature (°C)	TSS (mg/L)	CODcr (mg/L)	pH	NH3-N (mg/L)
WWTP1	109	3.2 (3.0, 3.4)	25.1 (21.2, 28.2)	297.0 (207.0, 376.0)	296.0 (227.0, 383.0)	7.4 (7.3, 7.5)	23.2 (16.4, 26.9)
WWTP2	109	16.4 (15.6, 17.4)	25.0 (20.8, 28.3)	343.0 (285.0, 412.0)	390.0 (326.0, 478.0)	7.2 (7.1, 7.3)	46.3 (41.4, 50.4)
WWTP3	101	8.6 (8.0, 9.8)	24.6 (21.3, 27.7)	229.0 (150.0, 306.0)	275.0 (202.0, 365.0)	7.3 (7.2, 7.4)	41.9 (36.5, 45.7)
WWTP4	103	63.5 (61.2, 66.2)	24.0 (19.9, 26.8)	238.0 (178.0, 286.5)	230.0 (182.5, 301.0)	7.3 (7.2, 7.4)	26.2 (23.1, 29.0)
WWTP5	104	13.7 (12.0, 14.8)	23.7 (20.5, 26.5)	294.0 (243.8, 353.5)	354.0 (303.8, 413.8)	7.3 (7.2, 7.4)	39.8 (37.0, 44.2)
Total	526	13.7 (7.7, 17.7)	24.4 (20.7, 27.6)	284.0 (207.0, 353.8)	316.5 (229.3, 392.0)	7.3 (7.1, 7.4)	35.5 (25.5, 44.2)

## Data Availability

The raw data supporting the conclusions of this article will be made available by the authors on request, as it involves certain information that is currently undisclosed.

## Supplementary Materials

The following supporting information can be downloaded at: https://www.mdpi.com/article/10.3390/v18050569/s1, Figure S1: Comprehensive regression diagnostics.

## Abbreviations

The following abbreviations are used in this manuscript:

SARS-CoV-2	Severe acute respiratory syndrome coronavirus 2
COVID-19	Coronavirus disease 2019
CODcr	Chemical oxygen demand
TSS	Total suspended solids
NH_3_-N	Ammonia nitrogen
GAM	Generalized additive models
RR	Relative risk
CIs	Confidence intervals
