# Supplementary figures and images for: Wastewater-Based Surveillance of SARS-CoV-2 for Early Warning of COVID-19 Infection Dynamics
Source: Viruses. 2026 May 18;18(5):569. doi: 10.3390/v18050569 (PMC13211602; doi:10.3390/v18050569)

# Supplementary Material

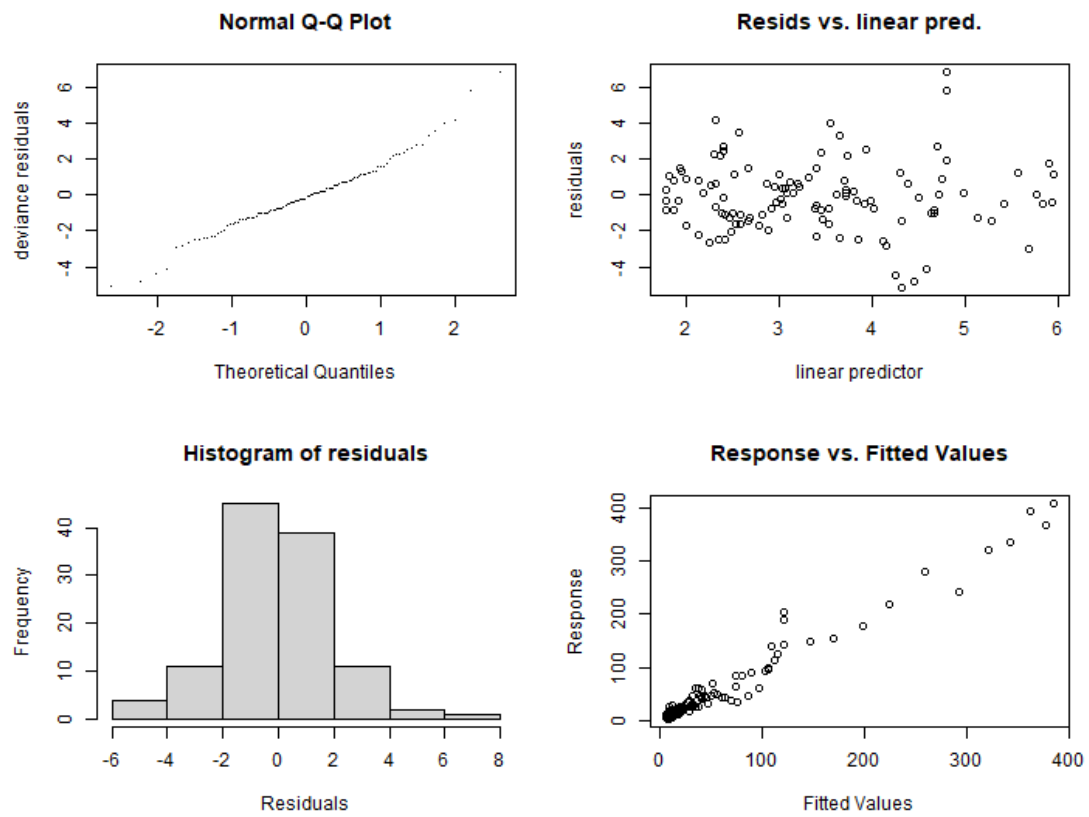

Figure S1. Comprehensive regression diagnostics.

Supplement: Supplementary file 1 [file viruses-18-00569-s001.zip › viruses-4273927-supplementary.pdf]
